# Estimating Tree Height-Diameter Models with the Bayesian Method

**DOI:** 10.1155/2014/683691

**Published:** 2014-02-25

**Authors:** Xiongqing Zhang, Aiguo Duan, Jianguo Zhang, Congwei Xiang

**Affiliations:** State Key Laboratory of Tree Genetics and Breeding, Key Laboratory of Tree Breeding and Cultivation of the State Forestry Administration, Research Institute of Forestry, Chinese Academy of Forestry, Beijing 100091, China

## Abstract

Six candidate height-diameter models were used to analyze the height-diameter relationships. The common methods for estimating the height-diameter models have taken the classical (frequentist) approach based on the frequency interpretation of probability, for example, the nonlinear least squares method (NLS) and the maximum likelihood method (ML). The Bayesian method has an exclusive advantage compared with classical method that the parameters to be estimated are regarded as random variables. In this study, the classical and Bayesian methods were used to estimate six height-diameter models, respectively. Both the classical method and Bayesian method showed that the Weibull model was the “best” model using data1. In addition, based on the Weibull model, data2 was used for comparing Bayesian method with informative priors with uninformative priors and classical method. The results showed that the improvement in prediction accuracy with Bayesian method led to narrower confidence bands of predicted value in comparison to that for the classical method, and the credible bands of parameters with informative priors were also narrower than uninformative priors and classical method. The estimated posterior distributions for parameters can be set as new priors in estimating the parameters using data2.

## 1. Introduction

Forests play a very important role not only in timber, mining, and recreational sectors, but also in global carbon cycles and climate change [1]. One of the most important elements of forest structure is the relationship between tree height and diameter. Individual tree height and diameter are the most commonly measured variables for estimating tree volume, site index, and other important variables in forest growth and yield, succession, and carbon budget models [2–4]. Tree diameter is relatively easy to measure accurately in the field at little cost. Conversely, tree height is not commonly measured for several reasons, which include (1) being time consuming to obtain; (2) chance of observer error; (3) visual obstructions [5]. Consequently, many foresters only subsample tree heights or do not measure heights at all. Often tree heights are estimated from observed diameter at breast-height (DBH) outside bark. The estimation of tree volume and site index, as well as the description of stand dynamics and succession over time, heavily relies on accurate height-diameter models [6]. A number of tree height-diameter models have been developed for various tree species [7–10]. These height-diameter models can be used to predict “missing” tree heights from measured DBHs [11, 12], indirectly predict height growth [13], and also estimate individual tree biomass using individual tree biomass equations [14]. Chave et al. [15] found that the most important parameters in predicting biomass of tropical forest tree species were in decreasing order of importance, diameter, wood density, height, and forest type (classified as dry, moist, or wet forest). The inclusion of height was reported to reduce the standard error of biomass estimates from 19.2 to 12.5%. Thus, accurate prediction of tree heights is essential for forest inventory, model simulation, and management decision making [2, 6].

Curtis [6] summarized a great many available height-diameter equations and used Furnival's index of fit to compare the performance of 13 linear functions fitted to second-growth Douglas-fir (*Pseudotsuga menziesii* (Mirb.) Franco) data. Since then, with the relative ease of fitting nonlinear functions, many nonlinear functions have been developed for height predictions [16, 17]. However, as tree form and allometry are influenced by both environmental and competitive factors [18–20], temporal changes in these conditions are likely to affect the height-diameter relationship. This may cause varied uncertainty in estimating height-diameter relationships at any given time. A major limitation of these equations is that they produce very different results when applied to different stands where the equations were originally developed [21, 22]. The height-diameter relationship is also not stable over time even within the same stand [23, 24]. Such differences could hold important implications for biomass and carbon storage potential.

This uncertainty resulting from temporal changes needs to be accounted for when interpreting height-diameter relationships in natural stands. Available methods do not apply to this problem. Bayesian inference is an alternative method of statistical inference that is frequently being used to evaluate ecological models [25–28]. In forestry, Bayesian methods have been adopted in several applications such as aboveground tree biomass [29], diameter distribution [30, 31], tree growth [32], individual tree mortality [33, 34], stand-level height and volume growth models [35, 36], and stand basal distribution [37]. Despite aforementioned studies, there is still a shortage of publications about application of the Bayesian methods in forestry, compared with other fields. Furthermore, to our knowledge, we found that there are no reports about the use of Bayesian methods in height-diameter curves.

In this study, we developed height-diameter models with nonlinear equations often used and selected the best nonlinear model for describing the height-diameter relationships. Based on the “best” model, we formulated a Bayesian modeling framework for exploring uncertainty of height-diameter relationships. Finally, we also compared the Bayesian method with classical method.

## 2. Data

The Chinese fir (*Cunninghamia lanceolata* (Lamb.) Hook.) stands are located in Fenyi County, Jiangxi Province, southern China. The longitude is 114°30′E, latitude 27°30′N. Mean annual temperature, precipitation, and evaporation are 16.8°C, 1656 mm, and 1503 mm, respectively.

**Figure 1:**
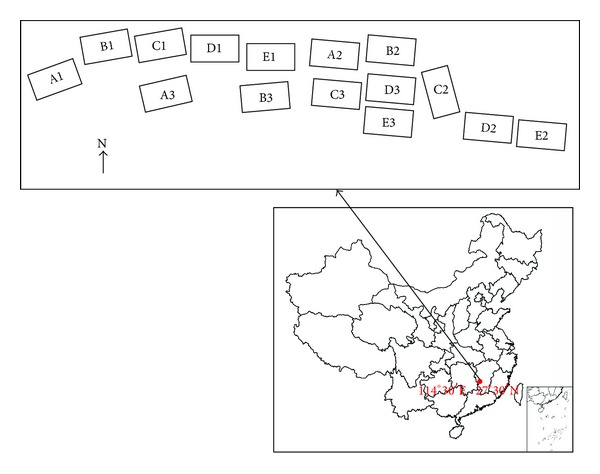
Layout of the sample plots for Chinese fir in this study.

**Figure 2:**
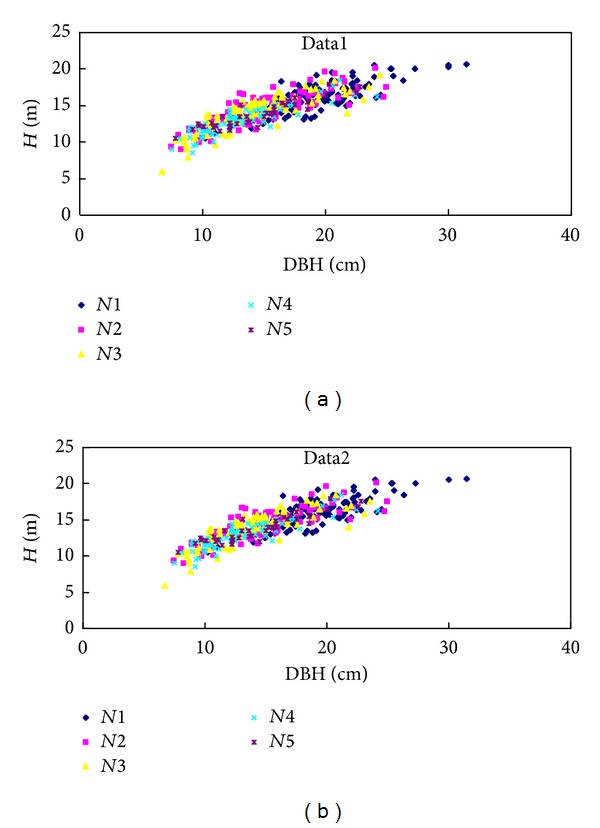
Scatter plot of tree height (*H*) against diameter at breast (DBH) for different densities. *N*1 = 2 m × 3 m (1667 trees/ha), *N*2 = 2 m × 1.5 m (3333 trees/ha), *N*3 = 2 m × 1 m (5000 trees/ha), *N*4 = 1 m × 1.5 m (6667 trees/ha), and *N*5 = 1 m × 1 m (10,000 trees/ha).

The plots were established in 1981, planted in a random block arrangement with the following tree spacings: *N*1: 2 m × 3 m (1667 trees/ha); *N*2: 2 m × 1.5 m (3333 trees/ha); *N*3: 2 m × 1 m (5000 trees/ha); *N*4: 1 m × 1.5 m (6667 trees/ha); *N*5: 1 m × 1 m (10,000 trees/ha). Each spacing level was replicated three times. Each plot comprised an area of 20 m × 30 m and a buffer zone of similarly treated trees surrounded each plot. Layout of the sample plots is shown in Figure 1. The tree diameter measurements in all of the plots were conducted after the tree height reached 1.3 m. More than 50 trees in each plot were tagged and measured for total height. Sampling was performed in each winter from 1983 to 1988 and then every two years until 2007. The forest structure of Chinese fir is stable when the forest is 25 years old. In this study, two data sets, data1 (24 years old) and data2 (26 years old), were used for modeling height-diameter relationships. The data1 was used for selecting the “best” model for analyzing the relationships of height-diameter and generating prior distributions of parameters for Bayesian method. The data2 was used for comparing the classical method with Bayesian method with uninformative priors and informative priors. The two data sets are described in Figure 2 and summary statistics are shown in Table 1.

## 3. Method

### 3.1. Base Height-Diameter Equations

Many nonlinear models have been used to model tree height-diameter relationships. Six nonlinear models (Table 2) were selected as candidate height-diameter models based on their appropriate mathematical features (e.g., typical sigmoid shape, flexibility) and possible biological interpretation of parameters (e.g., upper asymptote, maximum, or minimum growth rate) described in the literature [4, 38, 39].

### 3.2. Bayes' Rule

Let *y* = (*y*_1_, *y*_2_, *y*_3_, …) represent a vector of data and let *θ* = (*θ*_1_, *θ*_2_, *θ*_3_, …) be a vector of parameters to be estimated. Bayes' rule is then expressed as

(1) $$\begin{array}{c} p\left(y,\theta \right)=p\left(y\;|\;\theta \right)p\left(\theta \right)=p\left(\theta \;|\;y\right)p\left(y\right), \end{array}$$

where *p* represents the probability distribution or density function. Values for *θ* can be obtained by minimum least squares (MLS) or maximum likelihood estimation (MLE) in the classical approach. In the Bayesian framework, it uses probability distributions to describe uncertainty in the parameters being estimated. *θ* had a probability distribution that can be calculated as the rearranged form of (1):

(2) $$\begin{array}{c} p\left(\theta \;|\;y\right)=\frac{p\left(y\;|\;\theta \right)p\left(\theta \right)}{p\left(y\right)}, \end{array}$$

where *p*(*y*) = ∫*p*(*y* | *θ*)*p*(*θ*)*dθ* for continuous *θ*. Since it is the integration of admissible values of *θ*, *p*(*y*) does not depend on *θ* and can be viewed as a constant for fixed *y*, which yields the following [36]:

(3) $$\begin{array}{c} p\left(\theta \;|\;y\right)\propto p\left(y\;|\;\theta \right)p\left(\theta \right). \end{array}$$

We should note that the conditional distribution of *θ* given data *y* (*p*(*θ* | *y*)) is what we are interested in estimating and represents the posterior probability distribution (simply called posterior) in the Bayesian framework. *p*(*y* | *θ*) tells us the distribution of *y* assuming that *θ* is known, which is the likelihood function when regarded as a function of the parameters [40]. *p*(*θ*) is called the prior probability distribution for the parameters (simply called prior) and reflects information available about the hypothesis. Therefore, (3) indicates that the posterior distribution of *θ* is proportional to the likelihood of *y* given *θ* and the prior distribution of *θ*. The important characteristic of Bayesian method is that the parameters are treated as random variables [36, 41]. This is a very different assumption from that of classical method, which treats parameters as true, fixed (if unknown) quantities [40, 42].

### 3.3. Prior Distribution Specification

The choice of prior distribution is critical for Bayesian method [43]. In the above several nonlinear equations, we need to choose appropriate prior distributions for all parameters, including *a*, *b*, and *c*. Many researchers choose to use uninformative normal (Gaussian) priors that reflect prior “ignorance,” which would not have a strong influence on the parameters. Such priors typically arise in the form of a parametric distribution with large or infinite variance. Alternatively, if prior information is available from external knowledge (reported parameters from the literatures), this information can be used to construct a prior distribution. Often, there is little prior information regarding model unknowns, in which case an uninformative or vague prior distribution can be employed. In this study, we initially used uninformative for data1, Gaussian priors on all parameters (*a*, *b*, *c*): *a* ~ *N* (0, 1000), *b* ~ *N* (0, 1000), *c* ~ *N* (0, 1000). We set the previously estimated posterior distribution for data1 as the new prior distribution for data2.

### 3.4. Model Selection

The root mean square error (RMSE) was calculated for classical model performance evaluation. And deviance information criterion (DIC) was used to evaluate the Bayesian models. It is very useful in the Bayesian model selection [44]; DIC is characterized as

(4) $$\begin{array}{c} \text{DIC}=\text{Dbar}+\text{pD}, \end{array}$$

where Dbar refers to the posterior mean of the deviance and pD is the effective number of parameters in the model. The posterior mean of the deviance Dbar = *E*_*θ*_(−2log⁡(*p*(*y* | *θ*))) and pD = Dbar − Dhat. Dhat is a point estimate of deviance given by $\text{Dhat}=-2\log(p(y|\overset{-}{\theta }))$. As with RMSE, the model with the smallest DIC is selected to the “best” model.

In the Bayesian analysis, we set the previously estimated posterior distribution of parameters as the new prior distribution using data2. Mean relative deviation (MD), fit index (similar to *R*-square), and RMSE were used to compare the classical model with Bayesian model in the estimation stage. MD is given as

(5) $$\begin{array}{c} \text{MD}=\frac{1}{n}\overset{n}{\underset{i=1}{\sum }}\left({\hat{Y}}_{i}-Y_{i}\right), \end{array}$$

where *Y*_*i*_ represents the observed tree height of tree *i*, ${\hat{Y}}_{i}$ is the corresponding predicted value, and *n* is the number of observations.

Bayesian parameters were estimated using the WinBUGS version 1.4 [45], which implements Markov chain Monte Carlo algorithms using a Gibbs sampler [46]. Classical model parameters were estimated by use of the NLIN procedure (DUD method) in SAS.

## 4. Results

We set 300 000 iterations to run to ensure that maximum convergence and satisfied posterior distributions of estimated parameters for Bayesian method are obtained. Among those 300 000 iterations, the initial 20 000 iterations were discarded from analysis as burn-in iterations. To reduce the correlation between neighbouring iterations, the thinning parameters in the six models were all set to 3. After iterating, the mean, standard deviation (Std.), and 95% credible intervals with data1 can be obtained. The credible intervals of most parameters of the classical method were nearly equal to Bayesian method with uninformative priors (Table 3). In this study, based on RMSE (Table 3), we also found that Weibull model was the “best” model for describing height-diameter relationships of Chinese fir both for classical method and Bayesian method.

**Figure 3:**
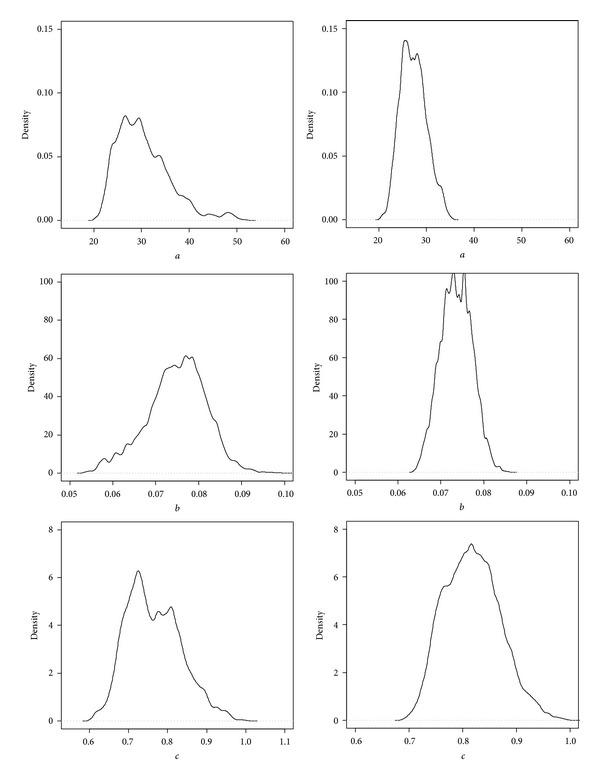
Posterior density curves of Weibull parameters based on Bayesian method with uninformative priors and informative priors using data2. The left row is for uninformative priors; the right row is for informative priors.

Based on the Bayesian method, the posterior probability distributions of the three parameters of Weibull model for data2 were obtained. The posterior probability distributions based on Bayesian method with informative priors were more concentrated than uninformative priors (Figure 3). Estimates of *a*, *b*, and *c* using Bayesian method and classical method were numerically identical in height-diameter model. The intervals of the three parameters estimates using classical method and Bayesian method with uninformative priors also had similar range, while they were wider than the intervals from Bayesian method with informative priors. The interval of asymptote parameter *a* using Bayesian method with informative prior was 68.4% narrower than the one of classical method, 66.7% narrower for *b*, and 59% narrower for *c* (Table 4).

**Figure 4:**
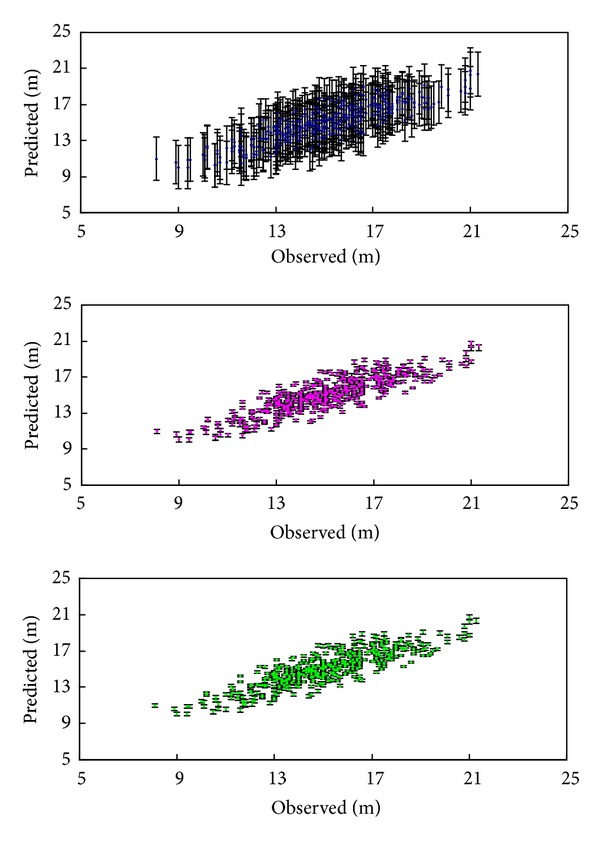
Predicted tree height values against observed values with data2. From up to down, the predicted values were obtained from classical method, Bayesian method with uninformative priors, and informative priors, respectively. The “error bars” were 95% confidence bands.

We also found that RMSE and fit index for Bayesian method and classical method were nearly equal, and Bayesian method with informative priors was slightly better than uninformative priors (Table 5). Despite the numerically equal evaluation statistics using classical method and Bayesian method, the credible bands of predicted values with Bayesian method were narrower than classical method, and the ones with informative priors were slightly narrower than uninformative priors (Figure 4).

## 5. Discussion and Conclusion

The curves generated with empirical models were checked with respect to their biological meaning; for example, height-diameter curves were assumed to demonstrate an approximately a sigmoid shape with clear inflection point that occurred in an early stage and other height increment should be more than zero [47]. In this study, the values of the evaluation statistics of the models showed that Weibull model most accurately estimated the tree height. Zhang [38] evaluated the prediction performance of six nonlinear height-diameter models for ten conifer species and found that Weibull function gave more accurate results than other model forms. Considering the model mathematical features, biological realism, and accurate prediction, we recommend the Weibull model as the base model in this case for further study.

Comparing the evaluation statistics from Bayesian method and classical method, we found that they were quite close (Table 5). However, we cannot only rely on the evaluations statistics for assessing a method, the prediction accuracy should also be taken into account. The improvement in prediction accuracy with Bayesian method led to narrower confidence bands of predicted value in comparison to that for the classical method (Figure 4). Bayesian method is an important statistical tool that is increasingly being used by ecologists [48, 49] and differs from classical method in main two ways. Firstly, Bayesian methods are fully consistent with mathematical logic, while classical methods are only logical when making probabilistic statements about long-run averages obtained from hypothetical replicates of sample data [50, 51]. Secondly, relevant prior knowledge about the data can be incorporated naturally into Bayesian analyses whereas classical methods ignore the relevant prior knowledge other than the sample data [41, 52]. Bayesian credible interval and classical confidence are usually numerically identical if the Bayesian prior is uninformative. An uninformative prior is one in which the data (by the likelihood, which is *p*(*y* | *θ*) in Bayes' rule) dominates the posterior, and the prior probabilities of all reasonable parameter values are approximately equal. Thus the posterior distribution has the same form as the likelihood. Since the posterior distribution with uninformative prior is less precise, the credible interval was wider (Table 4) [52].

With data2, the estimated posterior distributions for parameters can be treated as new priors in predicting the parameters. That is, informative new priors obtained from data1 are used in the estimation process of data2 to incorporate results from previously fitted models. It is the advantage of Bayesian method to update a model with new data. Therefore, not only are the data considered to be samples from a random variable, but the parameters to be estimated are regarded as random variables [40]. Thus, informative priors increased the precision of Bayesian estimates.

It is also noted that there are chances to improve the research. Additional variables, such as site index, age, and stand density, can be included in the analysis and develop hierarchical Bayesian models that can yield more accurate priors for new data. For example, it would be possible to develop procedures in which the prior information adapts to specific site and age. For more comprehensive and accurate relationships of height-diameter, additional variables describing stand density (e.g., stand basal area or number of trees) and site quality (e.g., site index) should be incorporated into the models [53, 54].

Height-diameter equations are crucial for estimating vertical forest structure [55], biomass, and carbon storage. Since collecting height data is costly and time consuming, the Bayesian method is valuable when data are limited, because it exploits prior information that can be obtained from other sources, for example, the fitted models, and it explicitly accommodates parameter variability. In conclusion, the Bayesian method is an alternatively feasible method for analyzing height-diameter relationships.

## Figures and Tables

**Table 1:** Summary statistics of tree diameter and height for two data sets.

Attributes	Data1 (24 years old, n = 445 trees)		Data2 (26 years old, *n* = 424 trees)	
	DBH	*H*	DBH	H
Min.	6.7	6	7.5	8.1
Max.	31.5	20.7	32.4	21.3
Mean	15.73	14.52	16.41	15.04
SD	4.28	2.39	4.40	2.37

SD: standard deviation.

**Table 2:** Nonlinear height-diameter equations selected in the study.

Model	References
Chapman-Richards: *H* = 1.3 + *a*(1 − exp⁡(−*b*DBH))^*c*^	[56]
Weibull: *H* = 1.3 + *a*(1 − exp⁡(−*b*DBH^*c*^))	[57, 58]
Logistic: *H* = 1.3 + *a*/(1 + bexp(−*c*DBH))	[58]
Gompertz: *H* = 1.3 + aexp(*b*/(DBH+*c*))	[59]
Bertalanffy: *H* = 1.3 + *a*(1 − exp⁡(−*b*DBH))^3^	[60]
Power law: *H* = 1.3 + *a*DBH^*b*^	[61]

**Table 3:** Parameter estimates of six models using classical and Bayesian methods with uninformative priors using data1.

		Classical method					Bayesian method				
Model		Parameter estimate		95% interval		RMSE	Parameter estimate		95% interval		DIC
		Mean	Std. error	Lower	Higher		Mean	Std.	Lower	Higher	
Chapman-Richards	*a *	22.95	2.472	18.10	27.81	1.1554	21.15	0.988	19.56	23.96	1398.34
	*b *	0.05	0.016	0.02	0.08		0.06	0.009	0.04	0.08	
	*c *	0.87	0.126	0.62	1.12		1.01	0.082	0.83	1.18	

Weibull	*a *	23.59	3.315	17.07	30.10	1.1551	26.58	4.619	20.86	38.67	1368.38
	*b *	0.07	0.006	0.06	0.08		0.07	0.005	0.06	0.08	
	*c *	0.90	0.100	0.71	1.10		0.85	0.080	0.71	0.99	

Logistic	*a *	19.12	0.661	17.82	20.42	1.1644	19.25	0.797	17.99	21.02	1406.18
	*b *	3.24	0.233	2.78	3.70		3.27	0.226	2.88	3.73	
	*c *	0.13	0.012	0.11	0.15		0.13	0.012	0.11	0.15	

Gompertz	*a *	28.53	2.322	23.97	33.10	1.1556	26.58	0.721	25.23	27.96	1399.03
	*b *	−15.48	3.258	−21.88	−9.08		−12.74	0.875	−14.38	−11.07	
	*c *	4.92	2.053	0.89	8.96		3.13	0.604	1.87	4.22	

Bertalanffy	*a *	16.58	0.164	16.26	16.91	1.2421	16.59	0.172	16.31	16.87	1462.49
	*b *	0.18	0.003	0.17	0.19		0.18	0.003	0.18	0.19	

Power law	*a *	2.78	0.122	2.54	3.02	1.1658	2.80	0.123	2.57	3.04	1406.01
	*b *	0.57	0.016	0.54	0.60		0.57	0.016	0.54	0.59	

**Table 4:** Parameter estimates of Weibull model using classical and Bayesian methods with data2.

Method		Parameter estimate		95% interval	
		Mean	Std. error	Lower	Higher
Classical method	*a *	29.14	8.446	12.54	45.74
	*b *	0.08	0.009	0.06	0.09
	*c *	0.77	0.116	0.54	1.00

Bayesian with informative priors	*a *	27.28	2.722	22.6	33.1
	*b *	0.07	0.004	0.07	0.08
	*c *	0.82	0.052	0.73	0.92

Bayesian with uninformative priors	*a *	30.41	5.56	22.59	44.84
	*b *	0.07	0.007	0.06	0.09
	*c *	0.76	0.070	0.65	0.92

**Table 5:** Evaluation statistics of classical and Bayesian method using data2.

Statistics	Classical method	Bayesian with uninformative priors	Bayesian with informative priors
MD	−0.0003	−0.0033	−0.0017
Fit index	0.7407	0.7407	0.7406
RMSE	1.2063	1.2063	1.2064
